# Copper Super-Dosing Improves Performance of Heat-Stressed Broiler Chickens through Modulation of Expression of Proinflammatory Cytokine Genes

**DOI:** 10.1155/2023/3559234

**Published:** 2023-09-13

**Authors:** Sudipto Haldar, Amrita Kumar Dhara, Sayantani Sihi Arora, Arpana Verma Mukherjee, Arup Nayak

**Affiliations:** Agrivet Research and Advisory Pvt Ltd., 714 Block A Lake Town, Kolkata 700089, India

## Abstract

Continuous exposure to high ambient temperatures brings about a number of oxidative damages in chickens. Copper (Cu), an active component of a number of antioxidative defence components, should arrest these changes to take place although that may not be possible under the standard dosing regimen followed by the industry. To ascertain the optimum dose response that may be beneficial in sustaining the performance of chickens under heat stress (HS), broiler chickens (*n* = 400) were exposed to high ambient temperature (between 27.2°C and 35.3°C) during 1–35 d. Copper (Cu) as Cu proteinate (Cu-P) at concentrations of 37.5, 75, 112.5, and 150 mg/kg was supplemented to the diet. The negative control (NC) diet did not contain any supplemental Cu. Increasing dietary Cu improved (*P* < 0.001) body weight, feed intake, and conversion ratio. Serum concentrations of total cholesterol at 21 d (*P* = 0.009), HDL cholesterol at 35 d (*P* = 0.008), LDL cholesterol at 21 d (*P* = 0.015), and triacylglycerol at both 21 d (*P* = 0.033) and 35 d (*P* = 0.001) decreased as Cu in the diet increased. As Cu in the diet increased, hemoglobin increased (*P* = 0.003) at 21 d, and the heterophil to lymphocyte ratio decreased both at 21 d (*P* = 0.047) and 35 d (*P* = 0.001). Superoxide dismutase and glutathione peroxidase activities increased when dietary Cu increased to 150 mg/kg (*P* < 0.01). Liver Cu at 35 d increased linearly with the dose of Cu in the diet (*P* = 0.0001). Selected bacteria were enumerated in the digesta to ascertain if Cu super-dosing affected their population in any way in the absence of any enteric challenge. *Escherichia coli* and total *Salmonella* numbers decreased (*P* = 0.0001), and total *Lactobacillus* increased (*P* = 0.0001) proportionately with dietary Cu. Interleukin-6 and tumour necrosis factor-*α* gene expression increased linearly (*P* = 0.0001) as Cu in the diet increased though the response plateaued at 112.5 mg/kg. It was concluded from the present experiment that during conditions of impending HS, dietary supplementation of 112.5 to 150 mg Cu/kg diet as Cu-P may be a novel strategy to alleviate the negative effects of HS without involving any apparent risk of Cu toxicity.

## 1. Introduction

High ambient temperature (HT) causes acute (a short and rapid increase in temperature) or chronic (extended exposure to moderately high temperature) heat stress (HS) and retards the performance of modern fast-growing broiler chickens leading to significant economic losses [1]. The intensive genetic selection for rapid growth has imparted higher metabolic rates in these birds and has made them less heat tolerant [2]. Oxidative damage of intestinal mucosa [3, 4] and change in small intestinal microbiota composition occur upon exposure to acute or even chronic HT, which not only impairs the general performance traits but also negatively affects the immune system in various ways, thus making chickens vulnerable to impending enteric and systemic diseases [5]. Chickens, like all other avian species, do not possess any functional heat dissipation mechanism except panting, and hence, when humidity in the environment increases concomitantly with temperature, the bird's core body temperature may exceed the upper critical limit to cause mortality [1, 2]. Climate-controlled houses play a crucial role in keeping the microenvironment surrounding the birds comfortable and conducive to optimum production and are one of the physical strategies employed worldwide to prevent HS-related mortality in broiler chickens. However, subtle physiological changes are inevitable even in environmentally controlled houses in areas experiencing high ambient temperature and humidity. These physiological changes may have negative impacts at the tissue and cellular levels, which may be sufficient to bring down the performance indices. Many dietary strategies, which are likely to mitigate the negative effects of HS by modulating the cellular and molecular functionality in commercially reared chickens, are being explored in recent times when owing to global warming, and maintaining the rearing temperature of a chicken-producing house within the thermoneutral zone has become a challenge. Trace minerals are one of the candidates which, when applied judiciously in the dietary regimen, may provide a solution to this problem amongst the trace minerals. The scope of copper (Cu) in sustaining the performance of chickens exposed to HS seems quite promising although this scope has not been hitherto researched to a great extent.

Dietary Cu requirement for broiler chickens varies from 8 mg/kg [6] to 16 mg/kg [7]. Adding Cu at higher concentrations in broiler diets for growth promotion has been reported [8]. Interestingly, such “more than the requirements” applications of Cu have not been extrapolated to study its effects in ameliorating HS. As an essential component of cytochrome oxidase, lysyl oxidase, superoxide dismutase, ceruloplasmin, and metallothionein, Cu maintains the redox balance and helps to achieve the targeted growth performances in poultry [9]. At an inclusion level of 150 to 200 mg/kg diet, Cu reportedly resulted in superior body weight (BW) and feed conversion ratio (FCR) in broiler chickens [10]. This supplementation strategy, which may be termed Cu super-dosing, should be a useful tool in sustaining the performance of domestic fowl, especially that of broiler chickens, under conditions of HS since Cu is an active component of the redox system of the body and considerable oxidative damages take place when a biological system gets exposed to elevated ambient temperature for a prolonged period. However, selecting the inclusion level of Cu should be performed carefully to prevent any possible toxicity since, as it will be discussed in more details in this study in a later section, Cu in higher inclusion levels may get accumulated in hepatic tissues and initiate oxidative damages by production of free radicals. This effect may vary with the source of Cu and hence is highly variable, and so far, no data are available with regard to Cu presented as yeast protein chelates.

In the present experiment, broiler chickens were exposed to naturally occurring cyclic HS from 1 to 35 d and were supplemented with Cu at graded levels up to 150 mg/kg diet as a yeast protein-Cu chelate (Cu-P). The experimental conditions simulated the practical situations often encountered by broiler operations, especially in tropical and subtropical countries, and paired controls for the heat-stressed groups needed to be improved. However, the basic objective of the experiment was to ascertain the effect of HT in broiler chickens when diets were near-deficient in terms of Cu and whether Cu super-dosing could alleviate the likely negative effects of HS. This design did not necessarily warrant heat-stressed paired control. The effects of Cu super-dosing on the expression of certain key immune-modulatory genes were also ascertained to assess if this strategy could help broiler chickens combat any impending diseases during exposure to HS.

## 2. Materials and Methods

### 2.1. Ethical Statement

The protocol was reviewed by the Institutional Animal Ethics Committee of the Research Station to ensure that the stipulations laid down by the Committee for the Purpose of Control and Supervision of Experiments on Animals (Ministry of Fisheries, Animal Husbandry and Dairying, Department of Animal Husbandry and Dairying, Government of India, 2020) were adhered to.

### 2.2. Experimental Design

The 35 d study was conducted with a flock of male broiler chickens (*n* = 400) fed a basal diet supplemented with a trace mineral premix devoid of Cu (negative control, NC). The NC diet was supplemented with Cu-P at varying levels to achieve the final Cu concentrations of 37.5, 75, 112.5, and 150 mg/kg, which corresponded to 250 (Cu-P 250), 500 (Cu-P 500), 750 (Cu-P 750), and 1000 (Cu-P 1000) mg/kg of the Cu-P in the diet. The customized Cu-P was manufactured in a commercial trace mineral production facility (Zeus Biotech P Ltd., Hebbal, Mysuru, India). The diets were adequate concerning all nutrients except Cu (Table 1) and did not contain any antibiotic growth promoter or probiotic. A phytase (*Buttiauxella* phytase with a declared activity of 5000 FTU/g, Danisco Animal Nutrition, Marlborough, UK) was used with its full matrix in all the diets. Diclazuril (0.5%) was added to prevent a possible coccidiosis outbreak. A yeast protein complex (similar to the one used to produce the Cu proteinate) was added to all the diets at the rate of 1 g/kg, and an equivalent amount of this was replaced when Cu-P was added to the diet. This was performed to ensure that the results obtained in the treatment groups were due to the effects of Cu only and not due to the yeast protein present in the Cu-P.

### 2.3. Bird Husbandry, Diet Preparation, and Measurement of Performance Traits

The experimental flock of one-day-old chickens (Vencobb 430 Y, Venkateshwara Hatcheries, Pune, India) was distributed in one of the five treatments following a completely randomized design. The chicks were weighed immediately after arrival and placed on a litter of fresh wood shavings and chopped paddy straw in individual pens (1.2 m × 1.2 m). A single pen constituted a replicate, and 8 such replicate pens were in a treatment group (10 chicks in a replicate and 80 chicks per treatment). The lighting period involved 23 h light during the first 7 days, followed by 20 h a day during the remainder. The experimental diets were produced by mixing all ingredients, excluding the Cu-P. This mix was then divided into 5 equal parts, and a premix (2 g/kg) containing Cu-P at variable quantities and finely ground maize (as filler) was added to each of these parts to achieve the desired levels of Cu in the corresponding treatment diets. The quantity of the maize was added in the above premix as filler was adjusted from the final formulation. The diets were pelleted in a prototype pellet machine (with a production capacity of 100 kg/h) at 80 ± 2°C at a steam pressure of 2.5 kg/cm^2^ with a dwelling time of 40 seconds (approximately) in the conditioner chamber. A two-phase feeding was performed in which the starter diet (1–21 d) was offered as crumbles, and the grower-finisher (22–35 d) diets were given as pellets. The pens were equipped with manually operated feeders and drinkers. Both feed and water were offered ad libitum. All diets were analysed for dry matter (method number 934.01), crude protein (method number 976.05), ether extract (method number 920.39), crude fibre (962.09), total ash (method number 942.05), and Cu [11]. The birds were vaccinated against infectious bronchitis (1 d), Newcastle disease (5 d and 20 d), and infectious bursal disease (12 d). Body weight (BW) was recorded pen-wise at 21 d and 35 d at the same time of the day (0800 h) without fasting. A measured quantity of feed was offered daily to each of the pens in two equal divisions. The cumulative feed intake (FI) was calculated during 1–21 d and 22–35 d by subtracting the quantity left in each pen from the total quantity of the feed offered during the aforementioned period. Average daily body weight gain (ADG), average daily feed intake (ADFI), and feed conversion ratio (FCR, calculated as ADFI: ADG) during 1–21 d, 22–35 d, and 1–35 d were calculated after adjusting the weight of the dead birds.

### 2.4. Induction of Heat Stress

Room temperature was maintained at approximately 35°C for at least 6 h a day (varying from 33 to 36°C) using an electrically operated brooding equipment. During the rest of the period in a day, the ambient temperature varied between 26 and 29°C since no environmental control system was made operational. High-velocity circulation fans were used to prevent humidity buildup in the room. The temperature and relative humidity of the room were recorded at hourly intervals with the help of data loggers installed at four corners of the experimental room and pooled daily.

### 2.5. Analyses of Serum Analyte and Haematological Parameters

At 21 d and 35 d, 1 bird was selected randomly from each of the replicate pens, and whole blood samples were collected from both the brachial veins. Blood samples from the right brachial vein were collected in vacutainer tubes without anticoagulant. The tubes were kept at room temperature to clot the blood, and the serum thus harvested was separated from the cells by centrifugation at 2500 rpm xg and stored in polystyrene tubes at −20°C until analysed for superoxide dismutase (SOD), glutathione peroxidase (GSH-Px), and malonaldehyde (MDA) by competitive enzyme-linked immune sorbent assays (ELISA) in a microplate reader (Biotek 800 TS absorbance reader, Agilent Technologies Inc., Santa Clara, CA, United States) using commercially available chicken-specific ELISA kits (Bioassay Technology Laboratories, Shanghai Korain Biotech Co., Ltd., Shanghai, China). A standard curve was generated using a polynomial quadratic regression equation tool (obtained from https://www.MyCurveFit.com) to derive the concentration of the respective biomarkers in serum. The sera were further tested photometrically for total cholesterol, high-density lipoprotein (HDL), low-density lipoprotein (LDL) cholesterol, and triacylglycerol using commercial kits (Transasia Bio-Medicals Ltd., India) in a semiautomated blood biochemistry analyser (RT-9100, Rayto Life and Analytical Sciences Co., Ltd., Shenzhen, China). Blood samples from the left brachial veins were collected in glass tubes containing EDTA as an anticoagulant. The tubes were handled with care to prevent damage to the blood cells. After blood sampling, a cellulose swab was pressed onto the wound until the bleeding stopped. Blood samples were brought to a laboratory near the broiler house for differential leukocyte counts. Blood smears were performed under dust-free and almost sterile conditions in the laboratory to achieve high-quality smears. Using a glass rod, one drop of blood was placed on a grease-free microscopic slide. The drop was spread on the glass slide by the wedge smear technique. The smears were air-dried, stained with Giemsa, and observed under the 40x objective of a trinocular microscope (Coslab 32 LED, Coslab India, Ambala, India). For the differential count, 200–400 leucocytes were determined. The H/L ratios were calculated by dividing the relative heterophil counts by the relative lymphocyte counts. Another aliquot of the fresh whole blood collected from the left brachial vein was used for the estimation of hemoglobin (Hb) by azide-methaemoglobin method in the blood analyser using a commercially available Hb kit (BeneSphera Hemoglobin Kit, Pennsylvania, USA).

### 2.6. Bacteria Enumeration in Ileal Digesta

At the end of the feeding trial, one bird from each of the replicate pens (*n* = 8 birds from a group) was selected randomly and slaughtered humanely (by inserting air emboli in the brachial vein). The small intestine was removed, and the tissue debris was washed off with sterile phosphate buffer saline (PBS). Ligatures were put on the Meckel's diverticulum and on the ileocecal junction with the help of sterile twines to prevent the mixing of caecal ileal contents. After repeated washing with sterile PBS, the ileum was separated, and the digesta present therein was emptied into autoclaved polystyrene tubes by applying gentle digital pressure. The tubes were stored at 4°C and, within 48 h, were cultured in specific media for enumeration of total *Salmonella* (HiTouch™ Salmonella Count Flexi Plate FL-023, for selective isolation and enumeration of *Salmonella typhi* and other *Salmonella* species; Hi Media Laboratories, Mumbai, India), *Escherichia coli* (Luria Bertani Agar, Miller or Miller Luria Bertani Agar, M1151, Hi Media Laboratories, Mumbai, India), and *Lactobacillus* spp. (Lactobacillus MRS Agar, M641, Hi Media Laboratories, Mumbai, India). All the cultures were incubated at 37°C for 36 h to 48 h to develop visible colonies. *Lactobacillus* culture was performed in the presence of 5% carbon dioxide. The number of visible colonies was enumerated manually under a colony counter, and the values were expressed in log_10_ colony-forming units (CFU) per g of ileal digesta [12].

### 2.7. Copper in the Liver

The whole liver from the slaughtered birds was removed after evisceration and washed with PBS to remove the tissue debris and blood. Tissue samples (approximately 5 g) were homogenized and dried at 80°C for 24 h in a hot air oven. The dried tissue samples were digested with concentrated nitric acid (HNO_3_) at 180°C. The digested samples were diluted with 1M HNO_3_ and used for analysis of Cu in a flame atomic absorption spectrophotometer [13].

### 2.8. Expression of Immune-Modulatory Genes

The expression of immune-modulatory genes, interleukin-6 (IL-6), and tumour necrosis factor alpha (TNF-*α*) was studied in hepatic tissues collected on 35 d. Tissue samples (100 mg) were preserved in RNAlater® (Sigma-Aldrich, Bangalore, India) at −20°C for later isolation of RNA with Trizol (Ambion, Thermo Fisher Scientific, Massachusetts, USA). The RNA was stored at −80°C in an ultra-low temperature freezer (U410–86, New Brunswick, Hamburg, USA). The purity and quantity of the RNA were determined by measuring absorbance in a NanoDrop One Microvolume UV-Vis Spectrophotometer (Thermo Scientific, Massachusetts, USA) at 260 and 280 nm while the integrity of the RNA was checked on 1.0% agarose gel using 1x TAE electrophoresis buffer. Complementary DNA (cDNA) was synthesised from the said RNA using a cDNA reverse transcription kit (QuantiTect Reverse Transcription kit, Qiagen Inc., Hilden, Germany) and stored at −20°C. The primer sets for the target genes and the housekeeping gene, beta-actin, were designed using the NCBI GenBank sequences (Table 2). Real-time PCR was performed in a 96-well microplates' Bio-Rad CFX-96 real-time PCR instrument (Bio-Rad Laboratories Inc., Hercules, California, USA) under the following conditions: 95°C for 3 minutes for initial denaturation, followed by 40 cycles of 95°C for 30 seconds, and at 60°C for 1 minute. Gene expression analysis was performed by the 2^−ΔΔ*Ct*^ method [14] in which the absolute and relative abundance of a particular immune modulatory gene was expressed as the cycle threshold (*Ct*) of the target genes and the fold chain increment (2^−ΔΔ*Ct*^) in the expression of the target gene in the treatment groups (in this case, the groups supplemented with 37.5 to 150 mg Cu/kg diet) relative to the untreated control group (in this case, the receiving no supplemental Cu). The latter was assigned a unit value of 1 (or 100%), and the values obtained with the other treatment groups were expressed relative to this unit value. The mean of qRT-PCR for a single biological replicate was the average of three technical replicates, and the mean was used as a single data point for calculation.

### 2.9. Statistical Analysis

All data were analysed by a general linear model of SPSS (version 26.1). Individual observations were the experimental units for performance traits, the pens, and the rest of the parameters. Results were presented as mean and pooled standard error of the mean. Probability values of *P* < 0.05 were considered statistically significant, and means were separated by Tukey's *B* test. Orthogonal polynomial contrasts were applied to determine the linearity of dose-response for Cu levels in the diet.

## 3. Results

### 3.1. Cu in Diet

The concentration of Cu (per kg fresh weight) in the Cu-P, the basal starter, and grower-finisher diets was 156 mg, 7.98 mg, and 7.03 mg, respectively. In the Cu-P supplemented diets, the concentration of Cu (mg/kg) was 48.65, 84.05, 121.0, and 162.55 for the starter and 49.85, 83.05, 119.95, and 159.4 for the grower-finisher phases in the Cu-P 250, Cu-P 500, Cu-P 750, and Cu-P 1000 groups, respectively.

### 3.2. Ambient Temperature

**Figure 1:**
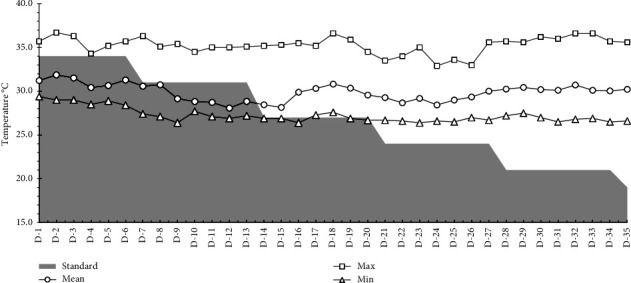
Maximum, minimum, and average daily temperature of the experimental room measured during the period of experimentation (1–35 d). Temperature was measured daily at hourly intervals and averaged on a 24 h basis to be plotted. The shaded area indicates the standard ambient temperature to be maintained for optimum production (Cobb Broiler Management Guide, 2013 available from http://www.cobb-vantress.com). The mean maximum temperature was 35.27°C (32.9–36.7°C), and the mean minimum temperature was 27.22°C (26.2–29.4°C).

Maximum temperature in the experimental room (Figure 1) varied between 33 and 36°C with an average of 35.3°C. The mean minimum temperature was 27.2°C (26.2 to 29.4°C). The mean temperature during 1–35 d was 29.7°C. Apart from a transient dip during 21–26 d, the maximum and minimum temperatures remained fairly constant. The relative humidity of the house (data not shown) varied in a day depending on the time of measurement, with relative humidity as high as 91% being recorded during the early morning. Relative humidity decreased in the daytime and went as low as 35%. During the overall period of 1–35 d, the average relative humidity of the experimental room was 65% (48–77%).

### 3.3. Performance Traits

Compared to the NC group, added Cu increased (Table 3) BW at 21 d (linear *P*=0.0001, quadratic *P*=0.013) and 35 d (linear *P*=0.0001), ADG during 1–21 d (linear *P*=0.0001, quadratic *P*=0.013), 22–35 d (linear *P*=0.029) and 1–35 d (linear *P*=0.0001), FI during 22–35 d (linear *P*=0.0001) and 1–35 d (linear *P*=0.0001), and FCR during 1–21 d (linear *P*=0.0001, quadratic *P*=0.004) and 1–35 d (linear *P*=0.0001, quadratic *P*=0.007). Liveability was similar across the groups. The mortality recorded during the study period was accidental, and necropsy examination did not reveal any pathognomonic lesion that could correlate with over-dosing of Cu in the dead birds.

### 3.4. Serum Analytes, Haematology, and Liver Cu Concentration

With an incremental dose of Cu in diet, serum concentrations of total cholesterol (linear *P*=0.009) and LDL cholesterol (quadratic *P*=0.015) decreased at 21 d, and that of HDL cholesterol decreased (quadratic *P*=0.008) at 35 d. Triacylglycerol was higher in the NC group at 21 d and 35 d, and Cu supplementation decreased it linearly at 21 d (*P*=0.033) and quadratically at 35 d (*P*=0.001). Hb increased (linear *P*=0.003) at 21 d, and heterophil decreased at 21 d (linear *P*=0.018) and 35 d (linear and quadratic *P*=0.001), while lymphocyte count did not change (*P* > 0.05) as dietary Cu levels increased. As a result, a wider H : L ratio in the NC group was observed compared to the treated groups at 21 and 35 d (*P* < 0.05). Amongst the Cu-supplemented groups, the ratio was narrower, with 150 mg Cu/kg diet at 21 d and 112.5 mg Cu/kg diet at 35 d (Table 4). The concentration of Cu in the liver (Table 4) at 35 d increased linearly with the dose of Cu (*P*=0.0001).

### 3.5. Bacteria in Ileal Digesta

The number of *E. coli* in ileal digesta decreased linearly (*P*=0.0001), while that of *Salmonella* decreased quadratically (*P*=0.0001) with the dose of Cu in the diet. *Lactobacillus* number increased when Cu was supplemented to the NC diet (*P*=0.0001, linear and quadratic).

### 3.6. Expression Analysis of Immune Modulatory Genes

Expression of the genes (Table 5) coding for IL-6 and TNF-*α*, expressed in terms of *Ct* target gene, increased linearly (*P* = 0.0001) as the dose of Cu increased in diet (Table 6) indicating a significantly high abundance of both the genes at Cu inclusion levels of 750–1000 mg/kg diet. The abundance of the said genes (Δ*Ct*) relative to the NC group also increased with the dose of Cu in diet (*P* = 0.0001 linear, *P* = 0.001 quadratic) for both IL-6 and TNF-*α*. The *Ct* values for the beta-actin gene, which served as the housekeeping gene, for both IL-6 and TNF-*α* remained unaffected by dietary Cu level (*P* > 0.05) and in both the analyses, which ran independently, and the *Ct* values of the beta-actin gene remained close to each other.

## 4. Discussion

The thermoneutral zones for broiler chickens are 28∼34, 25∼31, 22∼28, 20∼25, 18∼24, and 18∼24°C for each of the first six weeks of age, respectively [15]. So, it may be assumed that the birds in this experiment were under HS from 14 d of age onwards. This exposure to HS negatively affected overall BW and FCR, especially towards the terminal phase, and this happened irrespective of the level of Cu supplemented to the diets. The concentration of Cu in the NC diet was close to 8 mg/kg, which matches the NRC [6] recommended level. However, the data obtained in this experiment show that when the level of supplemental Cu as Cu-P was elevated to 37.5 mg/kg diet, the BW of the birds at the time of harvest increased by almost 4% over that in the NC group. In other words, a minimum of an additional 37.5 mg/kg Cu in the diet was required to reverse the detrimental effects of HS on performance, and it was possible even to achieve even a dose-dependent linear improvement in performance traits when the concentration of added Cu in the diet was increased up to 150 mg/kg diet. The present investigation adds to the existing data pool that dietary Cu super-dosing (as high as 125 to 150 mg Cu/kg diet) may improve BW, FCR [16–18], and nutrient digestibility [19] in adults as well as young chickens alike. The present data have corroborated the findings of El-Kassas et al. [20], who reported that dietary supplementation of Cu nanoparticles ameliorated the depression in BW and ADG observed in Ross and Cobb broiler chickens exposed to HS apparently due to a decreased FI [21] and suboptimal thyroid functioning [22] along with overt participation of corticosterone [23]. In this experiment, FI was largely similar across the treatments up to 21 d. Considering growth response as a function of FI, it may be assumed that dietary Cu concentration up to 8 mg/kg was insufficient to support normal growth and FCR during this period in the presence of moderate HS, which is plausible because Cu plays an important role in growth promotion [24], and energy digestibility [25]. The apparent Cu inadequacy in the NC group of birds failed to support the growth performance in the said group of birds because the negative effects of the dietary Cu inadequacy were exacerbated as the birds approached maturity in the presence of HS.

The concentration of blood metabolites in poultry may change upon exposure to HS [26, 27] due to oxidative damage in tissues [28, 29]. Acute HS reportedly elevated glucose and cholesterol in the serum of broiler breeders [27], while, in broiler chickens, a 23.9 to 37°C cyclic HS for four weeks increased glucose and lowered triglyceride and total cholesterol [26]. However, any such specific trend was absent in the present study indicating the subtle effect of HS and Cu super-dosing on the serum metabolites. It is important that both cholesterol and triacylglycerol decreased despite an elevated FI in the Cu-supplemented groups. It is plausible that superdosing of Cu improved utilization of nutrients at the tissue level, which, in turn, increased the cholesterol and triacylglycerol uptake from circulation into the tissue level, and this explains the comparatively better performance in the Cu supplemented groups even in the presence of HS. The activities of SOD, GPX, and MDA in serum were not affected by dietary Cu concentrations both at 21 d and 35 d, unlike other findings [27], and hence, it became apparent that Cu super-dosing in this experiment did not influence the oxidative properties of the experimental chickens. Circulatory oxidative enzyme activities vary a lot depending on the temperature and latency of heat exposure [4, 21, 27], and the present data did not give any clue about the effects of Cu super-dosing on oxidative damages in the experimental birds.

The dose-dependent increase in liver Cu concentration agreed with earlier findings obtained with Cu sulfate, tri-basic Cu chloride [17, 30], and Cu hydroxychloride [31] in broiler chickens. The available literature shows a strong correlation between the source of Cu and its assimilation rate in tissues [32], and abruptly increasing dietary Cu concentration may increase Cu concentration in excreta, thus raising serious environmental concerns. The increase in Hb due to Cu super-dosing was reported earlier [20] and suggests that the Cu from the yeast protein chelate was well assimilated. An elevated Hb might be correlated with a greater nutrient turnover rate, and it may be assumed that despite HS, Cu super-dosing sustained the pool of nutrients that bolstered the performance of the birds.

The H : L ratio, a widening of which is suggestive of a stressed condition in poultry [33], further indicated that Cu super-dosing ameliorated the HS in this experiment, with narrower ratios observed in all the Cu-supplemented groups. Considering H : L ratios of 0.2, 0.5, and 0.8 being indicative of low, optimal, and high degrees of stress in poultry, respectively [34], the stress was on the extremely higher side in the NC group, which came down to the level of “optimal” with 150 mg/kg Cu in the diet.

One of the intriguing qualitative parameters worth mentioning here is the reduction in the panting rate, especially towards the later age, in the Cu-supplemented birds. Panting and polypnea started from the 3rd week and occurred in all the dietary groups during the daytime when the temperature was at its peak, and the birds reverted to normal breathing activities when the room temperature was towards its lower side. The relative humidity of the experimental house was not too high, facilitating a quick reversal of the birds to normal breathing. The severity of panting increased further towards the terminal part of the experiment. Qualitatively, the reversal from panting to normal breathing occurred to a greater extent in the birds receiving Cu-P at higher doses, although owing to the visual nature of the observation, it was not possible to conclude about the exact inclusion level that resulted in reduced panting.

Exposure to HS brings about a profound shift in the neuroendocrine cascade in chickens, starting with the autonomic nervous system inducing tachycardia, panting, and enhanced blood flow towards the periphery to facilitate maximum heat loss to maintain body temperature [35]. Panting has far-reaching consequences. Owing to compromised splanchnic circulation, the muscle glycogen breaks down, and the capacity of muscles to store energy reduces [36, 37], thus disrupting the functioning of the electron transport chain. The electrons leak out from the mitochondria, generating reactive oxygen species [3, 38]. Cu is a pro-oxidant and scavenges the free radicals [39], being an integral part of the Cu-zinc superoxide dismutase [40], which is one of the main components of the antioxidant defence mechanism. It is claimed that Cu improves the efficiency of the electron transport system [41] and increases the antioxidant activities in fish [42]. In heat-stressed broiler chickens, dietary Cu supplementation as Cu-oxide nanoparticles decreased degenerative changes in hepatic tissues and modulated mRNA transcripts for heat shock protein, superoxide dismutase, and glutathione peroxidase [43]. In the present study, no oxidative stress biomarkers were affected by HS or level of Cu in diet, which is intriguing. With impending HS-induced oxidative damages, an upregulation in the expression of the nuclear factor (erythroid-derived 2) like −2 gene takes place that further triggers a variety of downstream target genes [44] which, in turn, through a series of rate-limiting reactions maintain glutathione and superoxide dismutase homeostasis [45]. In the present experiment, there was a numerical increment in the SOD at both 21 and 35 d, which suggests that the total antioxidant capacity increased by Cu super-dosing, although that of MDA is suggestive of little or no lipid peroxidation.

It should be noted that most stressor stimuli operate through an elevated corticosterone activity [22], again strongly controlled by the circadian rhythm [46]. Blood samples for the biomarker assays were collected in the morning when the room temperature was not still high enough to induce an overt HS reaction. As a result, the corticosterone-mediated stress responses possibly did not become strong enough to induce discernible changes in the circulatory concentrations of the biomarkers discussed previously.

The data related to the absolute number of bacteria in digesta suggested that there was an apparent shift in the numbers of bacterial species in the ileal digesta following Cu supplementation, which is in corroboration with the hypothesis that states that the growth-promoting effect of Cu is mediated through a modulated growth hormone action and a changed gastrointestinal microbiota [31]. An altered bacterial composition of the digesta with fewer potential pathogens such as *E. coli* and *Salmonella* decreases birds' susceptibility to diseases and reduces the recruitment of lymphocytes in protecting the intestinal tissues, which, in turn, might improve nutrient absorption [47]. The change in gut microbiota composition may be a direct effect of HS or may be a result of the altered immune profile in which the neuroendocrine behaviour shifts more towards corticosterone secretion during the periods of HS [48, 49], and it was apparent that due to graded level of Cu supplementation, the numbers of the potential pathogens decreased, and those of *Lactobacillus* increased. Inhibition of bacterial growth under the influence of Cu is highly dependent on the availability of free Cu in the media, and it is assumed that Cu salts with higher solubility should have greater bactericidal efficacy [50, 51], and the changes in bacterial numbers observed in the present study indicated that Cu from Cu-P was probably sufficiently available to bring about the desired changes in the ileal contents.

In human models, IL-6 serves as a pro-inflammatory gene during conditions of chronic inflammation, while during acute inflammation, it performs an anti-inflammatory action [52, 53]. Presumably, HS in the experimental birds might have disrupted the integrity of the mucosal barrier [54], which, in turn, facilitated the paracellular transport of endotoxins into the bloodstream leading to the activation of the innate immune system and systemic inflammation [36] and increased the expression of the IL-6 gene in liver coding for the proinflammatory cytokine. It is possible that the upregulation in the expression of the IL-6 gene in the liver due to Cu super-dosing happened under the influence of ceruloplasmin, which was expected to increase dose-dependent with Cu level in diet [37].

Scientific papers are ample to suggest that the negative effects of HS stretch beyond a depressed BW and FCR [55] but may extend to retarded growth of the digestive tract and disrupted the integrity of the mucosal barrier as well [54]. The latter, in turn, diminishes the innate immunity which allows pathogens to bind to and damage the intestinal epithelium [56] and facilitates the paracellular transport of endotoxins into the bloodstream leading to reactivation of the innate immune system and systemic inflammation [36]. The expression of the IL-6 gene, which increased as the dose of Cu increased, suggested that an oxidative damage might have taken place at the cellular level due to the HS, and this occurred irrespective of the level of Cu in the diet. As a matter of fact, several counter-current mechanisms operated on the experimental birds which not only confounded the data related to the expression of the concerned genes but at the same time also made them difficult to explain. For example, the possible oxidative stress mentioned previously might have had precipitated an innate immune response that increased the expression of the IL-6 gene which codes for the proinflammatory cytokine responsible for eliciting some adaptive immune responses and precipitates a condition termed as sickness behaviour [57]. Both Cu super-dosing and exposure to HS might increase ceruloplasmin in blood [58, 59] and elicit pro-inflammatory and antiinflammatory responses together which could increase the expression of IL-6 gene. As discussed earlier, the H : L ratio indicated that Cu super-dosing apparently decreased the level of stress on the birds in this experiment. Therefore, rather than the proinflammatory responses, it is the ceruloplasmin-mediated antiinflammatory pathway that increased the expression of the IL-6 gene in this case. This observation was, however, contradictory to an earlier study [37] which reported that the supplementation of 16 mg Cu per kg feed decreased liver concentration of IL-6 and TNF-*α*. The deficiency of Cu might delay the acute phase response because Cu is linked to synthesis of acute phase proteins such as ceruloplasmin, cytokine synthesis, and the generation of reactive oxygen species during phagocytosis [37, 60], and a moderate increment in the IL-6 and TNF-*α* activity was expected when Cu was supplemented at plethoric levels. Excessive Cu (as high as 330 mg/kg dry matter) reportedly caused pathologic changes and induced oxidative stress triggering NF-*κ*B pathway and induced inflammation through activation of proinflammatory programs [61–63]. Thus, it may not be inappropriate to assume that the beneficial effects of Cu super-dosing were partially negated by subtle toxic effects elicited in the experimental chickens. As a matter of fact, one of the concerns in designing the present experiment was the possibility of toxicity elicited by ingestion of almost 20 times more Cu than what has been suggested by NRC [6]. Dietary concentration of as high as 300 mg/kg Cu should cause growth depression Based on the current literature, Malinak et al. reported that dietary concentration of as high as 300 mg/kg Cu should cause growth depression [64] while that as high as 800 mg/kg may cause mortality [65, 66]. However, most of the toxicological data refer to the sulfate or oxide salts of Cu. Tribasic Cu chloride at more than 2000 mg/kg reportedly caused degenerative changes in gastrointestinal tract mucosa leading to high mortality in broiler chickens [64], but no such data are available for yeast protein chelates of Cu. Copper sulfate included at the rate of 300 to 450 mg/kg diet caused mild diarrhoea, anorexia, and reduced BW in some studies [30, 67] while many authors failed produce identical toxicological effects with similar or higher inclusion level of Cu in diets of broiler chickens [32, 68, 69]. Apart from the form in which Cu is presented, the other factor that influences Cu toxicity in cases of excessive intake is the solubility of the Cu salt at acidic and neutral pH. Copper as tri-basic Cu chloride (TBCC) is almost insoluble at neutral pH and at an acidic pH (in the presence of 0.1% HCl), and the solubility is reported to be approximately 76% in 1 h although Cu sulfate was almost completely soluble in both the pH [70]. This explains why more than 2000 mg/kg Cu in the form of TBCC was required to induce hepatic degeneration and gastrointestinal disorders in broiler chickens [64]. No such data are available for yeast protein chelates of Cu though so the data obtained with this experiment are going to be the first of its kind. Apparently, signs of toxicity were not evidenced in the experimental chickens in this study except for the BW gain and FCR getting plateaued beyond 112.5 mg Cu/kg diet despite a dose-dependent increase in Cu absorption up to 150 mg/kg Cu in the diet. Although, serum concentration of MDA, a key biomarker for cellular oxidative damages caused by super-dosing of Cu did not show any remarkable change with the dose of Cu in diet, considering the stalled performance indices, a subtle toxic effect beyond the inclusion level of 112.5 mg/kg could not be ruled out.

## 5. Conclusions

Dietary Cu at a concentration of 8 mg/kg, as suggested by NRC [6], was insufficient to support growth and feed efficiency under conditions of HT. Supplementation of 112.5 to 150 mg Cu/kg diet improved BW by 8% and FCR by almost 8 points in conditions of acute HS. Therefore, redefining the Cu requirement for modern broiler chickens, especially those likely to be exposed to environmental stresses, has become inevitable. Super-dosing the diet with as high as 112.5 to 150 mg Cu/kg as Cu-yeast-protein chelate may be a novel strategy to neutralize the negative effects of impending high ambient temperature without involving any apparent risk of Cu toxicity.

## Figures and Tables

**Table 1:** Composition (g/kg) and nutritive values (g/kg as fed, unless stated otherwise) of the basal diet.

Ingredients	Starter (1–21 d)	Grower (22–35 d)
Maize	541.0	577.0
Soybean meal^1^	288.0	240.8
Full fat soybean	65.0	70.0
Rice bran	30.0	30.0
Deoiled rape seed meal	25.0	25.0
Soybean oil	11.8	19.5
Dicalcium phosphate	15.5	14.0
Limestone powder	7.1	6.9
DL-Methionine	2.5	2.5
L-lysine HCl	1.4	1.8
L-threonine	0.4	0.7
Salt	3.0	2.5
Sodium bicarbonate	2.0	2.0
Trace mineral premix^2^	1.0	1.0
Choline chloride 60%	0.5	0.5
Vitamin premix^3^	0.5	0.5
Phytase 5000^4^	0.1	0.1
Coccidiostat (diclazuril 0.5%)	0.2	0.2
Yeast protein^5^	1.0	1.0
Deoiled rice bran^6^	4.0	4.0

*Nutritive values*		
AME MJ/kg	12.45	12.87
Crude protein^7^	228.6	208.3
Digestible amino acids		
Lysine	12.50	11.50
Methionine	4.80	4.40
Methionine + cysteine	8.80	8.40
Threonine	8.00	7.60
Tryptophan	2.00	2.00
Arginine	13.2	12.6
Isoleucine	8.9	7.9
Valine	9.8	9.1
Calcium^7^	10.12	8.40
Available phosphorus	4.5	4.2
Sodium	2.4	2.2
Potassium	9.0	8.9
Chloride	2.5	2.2
Choline mg/kg	1650	1550
Crude fibre^7^	31.17	28.21
Crude fat^7^	51.65	55.65

^1^Contained 490.5 g crude protein per kg; ^2^presented in the form of a protein chelate and contained (g/kg) iron (33.8), manganese (65.9), zinc (64.0), selenium (0.67), chromium (1.8), and iodine, as potassium iodide (4.28); ^3^Each kg contained vitamin A 13.5 MIU, vitamin D3 4.5 MIU, vitamin E 60 g, vitamin K3 3.5 g, vitamin B1 3.5 g, vitamin B2 8.0 g, vitamin B6 3.5 g, vitamin B 12 0.02 g, biotin 0.145 g, pantothenic acid 14.5 g, folic acid 2.25 g, niacin 60 g; ^4^*Buttiauxella* phytase with a declared activity of 5000 FTU/g; ^5^an equivalent amount was replaced with copper proteinate; ^6^used as a filler substance to accommodate the supplemental copper; ^7^analysed values.

**Table 2:** List of primers used for expression of interferon interleukin-6 (IL-6) and tumour necrosis factor alpha (TNF-*α*) genes.

Target genes	Accession number	Size (base pair)	Forward primer (5′–3′)	Reverse primer (5′–3′)
TNF-*α*	—	—	CAGCTGTGGGGAGAACAAAAGGA	TTGGCCCTTGAAGAGGACCTG
IL-6	AJ309540	254	CAAGGTGACGGAGGAGGAC	TGGCGAGGAGGGATTTCT
*β*-actin	—	—	GAGAAATTGTGCGTGACATCA	CCTGAACCTCTCATTGCCA

**Table 3:** Performance traits of broiler chickens supplemented with incremental doses of copper (as yeast protein) during 1–35 d^1^.

Parameters		Added Cu proteinate in diet mg/kg diet (added Cu mg/kg diet)					Pooled SEM	*P* value	
		0 (0)	250 (37.5)	500 (75)	750 (112.5)	1000 (150)		Linear	Quadratic
Body weight (g)	0 d	48.9	49.0	48.9	49.1	48.9	0.03	0.628	0.105
	21 d	969.2^a^	1029.4^b^	1048.0^b^	1053.1^b^	1069.6^b^	4.64	0.0001	0.013
	35 d	1878.4^a^	1958.3^ab^	1989.9^b^	2015.9^b^	2026.9^b^	9.25	0.0001	0.089

Gain (g/d)	1–21 d	43.82^a^	46.69^b^	47.58^bc^	47.81^bc^	48.6^c^	0.22	0.0001	0.013
	22–35 d	64.94^a^	66.34^b^	67.27^bc^	68.77^bc^	68.37^c^	0.64	0.029	0.537
	1–35 d	52.27^a^	54.55^b^	55.45^bc^	56.19^bc^	56.51^c^	0.26	0.0001	0.089

Feed intake (g/d)	1–21 d	58.3	58.7	59.5	59.5	57.7	0.28	0.689	0.474
	22–35 d	120.1^a^	122.3^ab^	122.6^ab^	123.7^ab^	126.9^b^	0.75	0.001	0.858
	1–35 d	83.0^a^	84.1^ab^	84.7^ab^	85.2^b^	85.4^b^	0.29	0.0001	0.819

Feed: gain^2^	1–21 d	1.330^c^	1.257^b^	1.250^b^	1.246^b^	1.189^a^	0.005	0.0001	0.004
	22–35 d	1.850	1.845	1.823	1.805	1.860	0.011	0.866	0.381
	1–35 d	1.589^b^	1.543^a^	1.528^a^	1.518^a^	1.512^a^	0.005	0.0001	0.007

Liveability (%)	1–35 d	96.25	100	97.5	97.5	98.75	0.64	0.585	0.644

^1^Means of 8 replicate pens/treatment with *n* = 10 birds/replicate at the beginning of the experiment; ^2^corrected for mortality; means with dissimilar superscripts in a row varied significantly.

**Table 4:** Serum analytes and haematological parameters at 21 and 35 d in broiler chickens supplemented with incremental doses of copper as (as yeast protein)^1^.

Parameters		Added Cu proteinate in diet mg/kg diet (added Cu mg/kg diet)					Pooled SEM	*P* value	
		0 (0)	250 (37.5)	500 (75)	750 (112.5)	1000 (150)		Linear	Quadratic
*Serum analytes (mmol/L)*									
Cholesterol	21 d	3.18^b^	3.11^ab^	2.99^ab^	2.83^ab^	2.68^a^	0.06	0.009	0.559
	35 d	4.36	4.01	4.42	3.67	4.37	0.09	0.258	0.818
HDL cholesterol	21 d	0.916	0.861	0.808	0.831	0.831	0.02	0.43	0.084
	35 d	0.870^c^	0.840^bc^	0.858^bc^	0.663^a^	0.781^b^	0.02	0.786	0.008
LDL cholesterol	21 d	1.46^b^	1.33^ab^	1.21^a^	1.28^ab^	1.27^ab^	0.02	0.139	0.015
	35 d	1.22	1.35	1.41	1.19	1.41	0.03	0.495	0.422
Triacylglycerol	21 d	0.985^c^	0.915^bc^	0.878^b^	0.878^b^	0.750^a^	0.03	0.033	0.851
	35 d	1.221^d^	0.911^bc^	0.966^c^	0.775^a^	0.859^b^	0.04	0.331	0.001
SOD (ng/mL)	21 d	1.356^a^	1.654^ab^	1.745^ab^	1.864^ab^	2.014^b^	0.052	0.001	0.359
	35 d	1.878^a^	2.075^ab^	2.24^ab^	2.415^ab^	2.614^b^	0.07	0.001	0.676
GSH-Px (ng/mL)	21 d	4.754	4.711	4.627	4.538	4.537	0.17	0.23	0.976
	35 d	0.949^a^	1.05^ab^	0.938^a^	1.174^ab^	1.32^b^	0.039	0.0001	0.429
MDA (nmol/mL)	21 d	3.230	3.125	3.061	2.995	2.971	0.053	0.422	0.976
	35 d	6.513	4.700	5.023	5.103	6.438	0.715	0.761	0.429

*Haematological parameters*									
Hemoglobin (g/dL)	21 d	14.92^a^	15.01^a^	15.27^ab^	17.43^c^	17.07^bc^	0.25	0.003	0.353
	35 d	17.57	18.21	19.31	18.41	18.13	0.33	0.686	0.317
Heterophil (%)	21 d	42.38^c^	39.88^b^	37.63^ab^	39.13^b^	36.88^a^	1.03	0.018	0.851
	35 d	76.13^a^	46.63^b^	31.38^a^	35.13^a^	48.25^b^	0.97	0.001	0.001
Lymphocytes (%)	21 d	59.88	61.63	60.13	61.75	62.88	1.03	0.625	0.723
	35 d	50.13	53.38	56.13	57.38	51.75	0.88	0.254	0.096
H : L ratio	21 d	0.723^b^	0.652^a^	0.650^a^	0.640^a^	0.606^a^	0.024	0.047	0.953
	35 d	1.52^c^	0.895^b^	0.558^a^	0.621^a^	0.953^b^	0.02	0.0001	0.0001
Liver Cu (mg/kg DM)	35 d	12.00^a^	15.41^ab^	16.45^ab^	19.58^bc^	23.54^c^	0.485	0.0001	0.646

^1^There were 8 replications/treatment with *n* = 10 birds/replication. Samples were collected from a single bird selected randomly from a replicate. Means with dissimilar superscript in a row varied significantly.

**Table 5:** Relative expression of genes coding for interleukin-6 and tumour necrosis factor alpha at 35 d in hepatic tissues of broiler chickens supplemented with incremental doses of copper (as yeast protein)^1^.

Parameters	Added Cu proteinate in diet mg/kg diet (added Cu mg/kg diet)					Pooled SEM	*P* value	
	0 (0)	250 (37.5)	500 (75)	750 (112.5)	1000 (150)		Linear	Quadratic
*Interleukin-6*								
*Ct*-target gene	36.05	33.15	32.07	29.59	28.31	0.16	0.0001	0.174
*Ct*-actin	19.86	20.25	20.12	20.07	19.68	0.11	0.493	0.196
∆*Ct*-target gene	16.19	12.89	11.94	9.51	8.62	0.17	0.0001	0.022
Relative fold chain (2^∆*Ct*^)	1.00	9.91	20.03	103.73	193.89	3.56	0.0001	0.001

*Tumour necrosis factor-α*								
*Ct* target gene	38.09	35.97	33.64	31.04	30.24	0.13	0.0001	0.03
∆*Ct*-actin	20.41	20.38	20.46	19.82	19.74	0.12	0.139	0.397
∆*Ct*-target gene	18.44	15.59	13.18	11.22	10.5	0.12	0.0001	0.0001
Relative fold chain (2^∆*Ct*^)	1.00	7.54	46.11	183.93	246.46	6.05	0.0001	0.0001

^1^There were 8 replications/treatment with *n* = 10 birds/replication. Samples were collected from a single bird selected randomly from a replicate. Means with dissimilar superscript in a row varied significantly.

**Table 6:** Selected bacteria numbers (log_10_ colony-forming units/g digesta) at 35 d of age in ileal digesta of broiler chickens supplemented with incremental doses of copper (as yeast protein).

Parameters	Added Cu proteinate in diet mg/kg diet (added Cu mg/kg diet)					Pooled SEM	*P* value	
	0 (0)	250 (37.5)	500 (75)	750 (112.5)	1000 (150)		Linear	Quadratic
*Escherichia coli*	9.01^c^	8.95^c^	8.70^b^	8.60^ab^	8.51^a^	0.02	0.0001	0.001
*Salmonella* spp.	8.76^b^	8.62^ab^	8.55^a^	8.53^a^	8.60^ab^	0.03	0.201	0.0001
*Lactobacillus* spp.	8.77^a^	8.93^b^	8.96^b^	9.03^b^	9.00^b^	0.01	0.0001	0.0001

^1^There were 8 replications/treatment with *n* = 10 birds/replication. Samples were collected from a single bird selected randomly from a replicate. Means with dissimilar superscript in a row varied significantly.

## Data Availability

The data used to support the findings of this study are included within the article. Further access to the raw data is possible on request. The corresponding author should be contacted to have access to the raw data.
